# How Can *PLOS Computational Biology* Help the Biological Sciences?

**DOI:** 10.1371/journal.pcbi.1003262

**Published:** 2013-10-03

**Authors:** Ruth Nussinov

**Affiliations:** 1 Basic Science Program, SAIC-Frederick, Inc., Cancer and Inflammation Program, National Cancer Institute, Frederick, Maryland, United States of America; 2 Sackler Institute of Molecular Medicine, Department of Human Genetics and Molecular Medicine, Sackler School of Medicine, Tel Aviv University, Tel Aviv, Israel

A year has passed since I took over as the Editor-in-Chief of *PLOS Computational Biology*, making it a good time to reflect on the journal, reassess the needs of our community, and, broadly, the direction of computational biology within the framework of the biological sciences. The rapid growth in computational power, the masses of data that need to be understood, and the advances in the biological sciences all point to a need to reevaluate directions, merging these with the fast pace of discoveries made by experimental studies. Are we, as computational biologists, contributing as much as we can to the advancement of the sciences in key areas? Since *PLOS Computational Biology* is a broad, community-based open-access journal, the areas of focus that we deem central to the field may impact the future of computational biology.

The biological sciences increasingly shift to research projects that aim to understand the causes and the mechanisms of diseases and to discover therapeutics. This shift over recent years is fueled both by the desire to alleviate human suffering and by the preferences of funding agencies. Within this framework, computational biology can effectively contribute to the understanding of a broad range of biological processes under normal physiological conditions and in disease; and it can do this across a range of scales, from the molecular and biochemical to the organismal and population levels. As a leading journal in the computational sciences, *PLOS Computational Biology* can, and, I believe, should, foster a highly stimulating and interactive environment among computational and experimental scientists in our community toward these aims.

Computational biology increasingly gains the scientific center stage. It is an exciting interdisciplinary field that draws scientists from different fields: physics, chemistry, mathematics, engineering, computer science, and biology. Computational biology aims to organize and make sense of huge amounts of data and large-scale cellular processes and pathways, and, at the same time, to understand biological phenomena on the atomic scale. Experiments obtain information at multiple levels. Computational biology is a quantitative field. The goals of computational biology are to distinguish between noise and signals, obtain and quantify trends, and put these together, so that we are able to figure out how the information flows, how the processes are regulated, and what goes wrong in disease. We would like to understand the mechanisms through which mutations lead to cell proliferation, how the signals transmit in the cellular network and how external stimuli translate to cellular differentiation and to turning genes on and off, and how viruses enter cells. However, beyond these, computational biology aims to use the information to make predictions and obtain experimentally testable models.

*PLOS Computational Biology* embraces all areas of computational biology. However, I believe that to help the advancement of the biological sciences, the journal should consider papers that address biologically relevant questions that are of broad interest and provide new concepts that can guide the design of experiments. This has been in our scope since the inception of the journal, and we shall continue to follow these guidelines. It is emboldening to think of open questions in the biological sciences, particularly those relating to diseases, where computational biology can drive progress.

*PLOS Computational Biology* is highly regarded by the scientific community. We should aim to not only retain this appreciation but to further it. In response to requests by our community, the first step that I took after taking over as the Editor-in-Chief was to introduce a Methods section, led by Thomas Lengauer. Under Thomas' leadership, this section is flourishing, and is fast becoming highly popular, with an increasing number of submissions. The section strives to publish only the most outstanding methods, aiming to consider only those that are of exceptional quality. We are now also actively engaged in reducing the response times to authors, publication processing, and in simplifying the submission process.

The content of *PLOS Computational Biology* is important not only for scientific advancement; it also bears on our students who should be exposed to a variety of ways to define, analyze, question, and solve relevant scientific research problems computationally, relying on experimental data. For *PLOS Computational Biology* to help and inspire the biological sciences, we, as a community, should remain tuned-in to research trends and new data, and take action. These goals are shared with the *International Society for Computational Biology* (ISCB), with whom we are proud to partner. Together, we hope to make a difference.

Finally, the achievements of *PLOS Computational Biology* would not have been possible had it not been for the devoted journal staff, who are always there to help, guide, suggest, and follow on initiatives, and our extended Editorial Board. Armed with these, and embracing our community, our open-access *PLOS Computational Biology* journal will continue to thrive.

